# Supplementary material for: Two Wheat Cultivars with Contrasting Post-Embryonic Root Biomass Differ in Shoot Re-Growth after Defoliation: Implications for Breeding Grazing Resilient Forages
Source: Plants (Basel). 2019 Nov 2;8(11):470. doi: 10.3390/plants8110470 (PMC6918441; doi:10.3390/plants8110470)
Supplement: Supplementary file 1 [file plants-08-00470-s001.pdf]

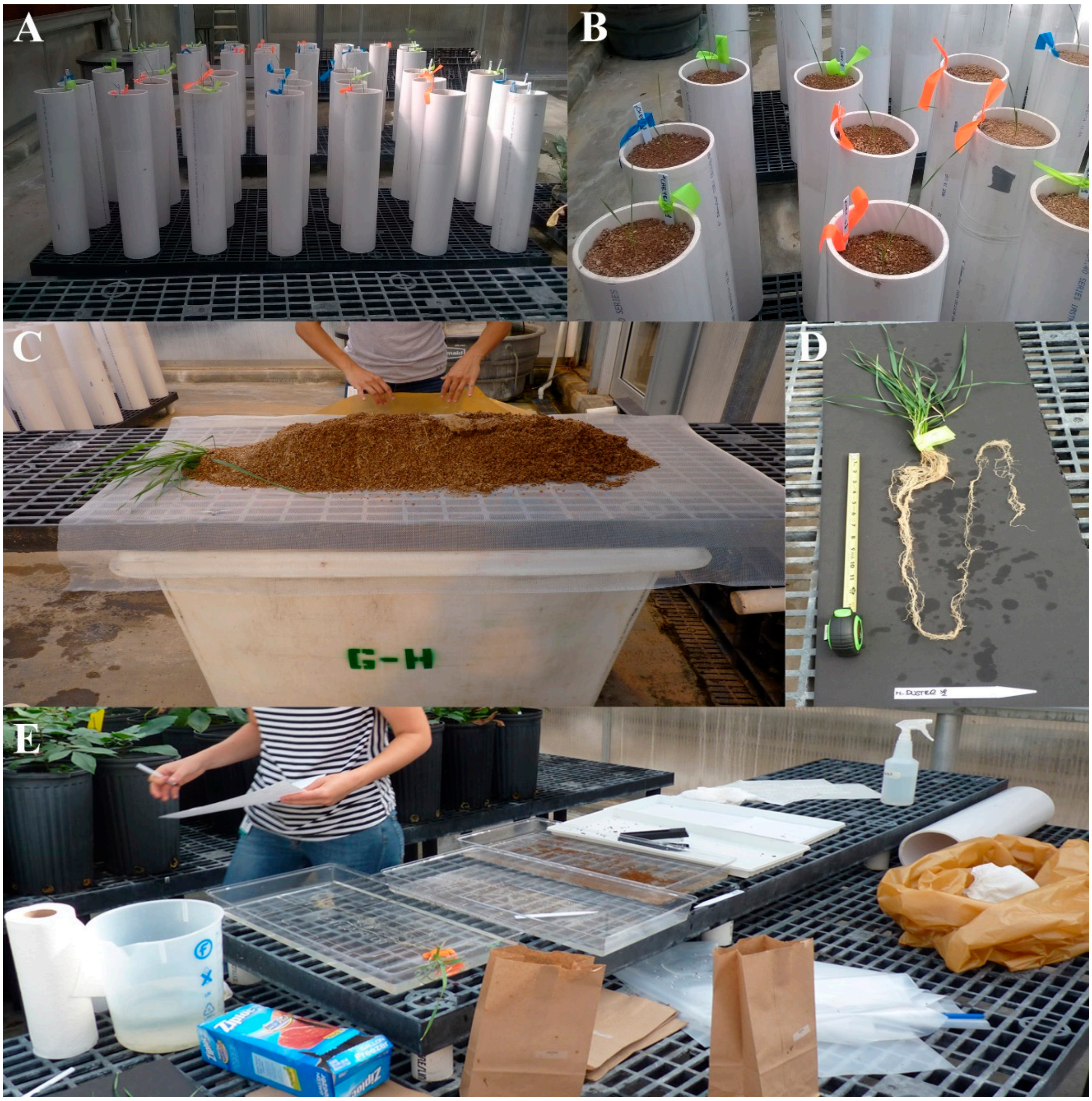

Figure S1. Methodology for root and shoot phenotyping. (**A**) System of forty-two pipes stablished in the greenhouse. Each pipe hosted one individual wheat seedling. (**B**) Individual pipes were labelled with the name of the cultivar and the cutting treatment applied. After ten weeks of growth, plants were removed from the pipes and the soil mixture was washed from the roots (**C**). **(D)** Individual plants were photographed and processed to obtain root and shoot measurements. (**E**) Detailed root washing station. Different root and shoot traits were quantified, and any remaining turface-sand mixture was washed from the roots..
